# Potential therapeutic role of SIRT1 in age- related hearing loss

**DOI:** 10.3389/fnmol.2022.984292

**Published:** 2022-09-20

**Authors:** Tingting Zhao, Guangyong Tian

**Affiliations:** ^1^Department of Otorhinolaryngology-Head and Neck Surgery, The Third Affiliated Hospital of Southern Medical University, Guangzhou, China; ^2^Guangdong Provincial Key Laboratory of Bone and Joint Degeneration Diseases, Guangzhou, China

**Keywords:** SIRT1, age-related hearing loss, ARHL, cochlea, hearing loss, age

## Abstract

Age-related hearing loss (ARHL) is a major public health burden worldwide that profoundly affects the daily life of elderly people. Silent information regulator 1 (SIRT1 or Sirtuin1), known as a regulator of the cell cycle, the balance of oxidation/antioxidant and mitochondrial function, has been proven to have anti-aging and life-extending effects, and its possible connection with ARHL has received increasing attention in recent years. This paper provides an overview of research on the connection between SIRT1 and ARHL. Topics cover both the functions of SIRT1 and its important role in ARHL. This review concludes with a look at possible research directions for ARHL in the future.

## Introduction

### Age-related hearing loss

Hearing loss, in its variants conductive, sensorineural or mixed, may be classified according to its etiology as hereditary, traumatic, environmental, and induced by drugs (Cunningham and Tucci, 2017). According to the WHO 2021 World Report on Hearing, hearing loss currently affects approximately 1.6 billion people worldwide, or 20.3% of the global population, and more than 5.5% (430 million) of the world’s population have moderate or higher hearing loss. Globally, hearing loss is the third leading cause of disability (GBD 2019 Diseases and Injuries Collaborators, 2020; Tordrup et al., 2021). The number of people with hearing loss is expected to reach nearly 250 million by 2050. Unresolved hearing loss costs more than $980 billion annually globally (World Health Organization [WHO], 2021). WHO is scaling up health interventions for the ear and hearing, and expects to avert more than 130 million disability-adjusted life years over the next decade. These gains translate into a monetary value of more than $1.3 trillion. In addition, investments in hearing care will result in more than $2 trillion in productivity gains worldwide by 2030 (Tordrup et al., 2021). Therefore, it is significant to intervene and research on hearing loss.

Age-related hearing loss (ARHL), also known as presbycusis, is a progressive decline in hearing sensitivity and language comprehension that is associated with aging. Therefore, this can seriously affect the social function and mental health of the elderly individuals (Bowl and Dawson, 2019; Noble et al., 2022). Current research suggests that ARHL can promote the development of cognitive dysfunction (Loughrey et al., 2018; Golub et al., 2020). As the population ages, the prevalence of ARHL will continue to increase, which will greatly increase the socioeconomic burden (Jafari et al., 2019). Therefore, research on ARHL is urgently needed.

Hearing, as people’s internal sense, is important for daily communication, and hearing impairment can greatly affect social function and mental health. Hearing impairment can seriously affect people’s social functioning and social health. It is now generally accepted that hearing loss is an independent risk factor for cognitive dysfunction (Golub et al., 2020; Lozupone et al., 2020). There are several hypotheses to explain the potential relationship between auditory and cognitive impairment; one hypothesis is that is that the relationship is underpinned by general neurodegeneration in aging, while the other hypothesis suggests that auditory impairment and sensory deprivation are causally linked to cognitive impairment (Slade et al., 2020). At present, whether the two are causal or mutually reinforcing needs to be further verified. With the progress of population aging, the prevalence of ARHL will continue to increase in the future, which will greatly increase the socioeconomic burden.

The inner ear pathology of ARHL is characterized by loss of inner and outer hair cells, vascular stripe atrophy, loss of spiral ganglion cells, and degeneration of spiral ligaments (Bowl and Dawson, 2019). It is currently believed that ARHL can be caused by deafness-related genes, noise pollution, and other single or interactive effects, while the accumulation of chronic oxidative stress damage with aging is likely to be a major cause of ARHL (Fujimoto and Yamasoba, 2014). The C57BL/6J mouse is an excellent animal model for studying ARHL. C57BL/6J mice show hearing loss with age, with elevated hearing thresholds starting at 6 months or even as early as approximately 3 months of age (Hequembourg and Liberman, 2001) and a linear trend of increasing hearing thresholds as time progresses (Ison et al., 2007).

Comparing human glucose-6-phosphate dehydrogenase (G6PD) gene-transferred C57BL/6 mice with wild-type mice, G6PD overexpression was found to enhance antioxidant capacity, attenuate oxidative damage and delay the onset of hearing loss (Bermúdez-Muñoz et al., 2020). p43 knockout mice exhibited more severe and earlier onset of hearing loss with age progression than wild-type C57BL/6 mice, precisely because of mitochondrial dysfunction and increased oxidative damage due to p43 gene deletion (Affortit et al., 2021). Mitochondrial dysfunction and oxidative stress are common features in neurodegeneration and aging (Giordano et al., 2013; Fang et al., 2014), now evidence indicates that the dysregulation of glial cells (e.g., satellite cells) and degeneration of the ganglion node structure are important mechanisms of ARHL (Panganiban et al., 2022). These studies suggest that a strong antioxidant function plays an important role in delaying ARHL. Mitigating oxidative damage and restoring the balance of the oxidative/antioxidant system are major hot spots in current research on the prevention and treatment of ARHL. Therefore, SIRT1, which has good antioxidant, anti-aging, and neuroprotective effects (Giordano et al., 2013), is receiving increasing attention in the study of ARHL.

### Silent information regulator 1

SIRT1, a nicotinamide adenine dinucleotide (NAD+)-dependent class III histone deacetylase, is associated with lifespan extension. It is known for its ability to balance oxidation/antioxidation and reduce mitochondrial damage, DNA damage and apoptosis (Chen C. et al., 2020). It has been found to play a non-negligible role in ARHL (Salam et al., 2021).

### Silent information regulator 1 in the inner ear

SIRT1 is widely distributed in various organs and tissues throughout the body and is involved in anti-aging, anti-inflammatory, antioxidant, and cell cycle regulation in the brain, liver and other important organs (El Hayek et al., 2019; de Gregorio et al., 2020; Zhang X. S. et al., 2021; Kadono et al., 2022). It has been confirmed that SIRT1 is also present in the cochlea (Xiong et al., 2014). In the mouse cochlea, SIRT1 was expressed in the inner and outer hair cells, marginal, and intermediate cells of the vascular striae, spiral ligament, and spiral ganglion cells. Immunofluorescence and histochemical results showed that SIRT1 was expressed in the nuclei of the above cells, and the expression in aged mice was significantly lower than that in young mice (Chang et al., 2018; Pang et al., 2019). The results confirmed that SIRT1 is strongly associated with ARHL and deserves in-depth study by audiologists.

SIRT1 plays a crucial role in hearing loss, especially ARHL. This review elucidates the structure and function of SIRT1 and its localization in the cochlea. Moreover, it mainly explains the relationship between SIRT1 and ARHL, as well as the current research progress related to SIRT1 and the prevention or treatment of ARHL, and provides our own views on future treatment and research strategies for ARHL.

## Structure and function of silent information regulator 1

Sirtuins are (NAD +)-dependent class III histone deacetylases. The family consists of seven members in mammals (SIRT1, SIRT2, SIRT3, SIRT4, SIRT5, SIRT6, and SIRT7). These family members are characterized by the possession of the sirtuin core structural domain (Frye, 1999). Sirtuins are associated with many pathophysiological activities, such as genome stabilization, cancer, oxidative stress response, apoptosis, metabolism, aging, proliferation, and inflammation (Bonkowski and Sinclair, 2016). SIRT1 is the first to be identified and is more recognized in the sirtuins family (Haigis and Sinclair, 2010). In mammals, SIRT1 is composed of 747 amino acids and includes three regions: the central core (273–517 amino acids), which possesses the structural domain of the deacetylase; the N-and C-terminal regions, which are located on either side of the enzyme core; and the catalytic domain, which consists of 250 amino acids and is highly conserved among species (Sauve et al., 2006). SIRT1 controls DNA transcription by transferring acetyl groups from ε-N-acetyllysine amino acids to histones of DNA (Maiese, 2021). It has been suggested that SIRT1 is a possible substrate for autophagy. SIRT1 is mainly located in the nucleus, but also in the cytoplasm and mitochondria, and is closely associated with senescence when it is present in the cytoplasm (Xu et al., 2020). Silent information regulator 2 (Sir2) is a mammalian homolog highly similar to SIRT1 (Smith, 2002). In 1999, it was found that the lifespan of Sir2 mutant yeast cells was significantly shorter, while the lifespan of yeast overexpressing Sir2 was longer than that of wild type yeast (Kaeberlein et al., 1999). SIRT1 was also found to extend the lifespan of *Cryptobacterium hidradenum* (Tissenbaum and Guarente, 2001), mice (Satoh et al., 2013), and flies (Rogina and Helfand, 2004); this effect was associated with caloric restriction, which means that caloric restriction activates SIRT1 to exert lifespan-extending effects.

**FIGURE 1:**
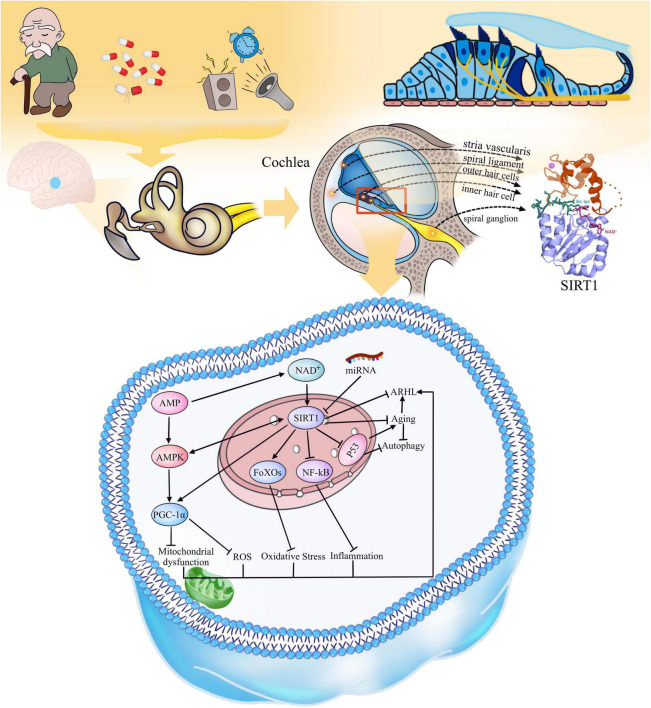
Aging and environmental factors lead to the decline of auditory center and inner ear functions, resulting in ARHL. **Distribution of SIRT1 in cochlea:** SIRT1 is expressed in inner and outer hair cells, spiral ligament, margin cells and intermediate cells of stria vascularis, and spiral ganglion cells of cochlea. **The function of SIRT1:** SIRT1 is regulated by NAD +, AMPK is regulated by AMP, and AMPK and SIRT1 can promote each other, while some specific miRNAs can inhibit the activity of SIRT1. SIRT1 can activate PGC-1α and FoXOs, enhance the resistance to oxidative stress damage, keep the normal function of mitochondria; SIRT1 can also deacetylate to reduce the activity of NF-κB, p53 and other inflammatory and aging-related factors to reduce their activity and promote the increase of autophagy. As a consequence, SIRT1 can delay the aging and age-related hearing loss.

The deacetylation of SIRT1 reverses the acetylation of lysine residues on its target proteins by hydrolyzing an NAD+ and generating nicotinamide and a unique metabolite called 2′-O-acetyl-ADP-ribose (Hwang et al., 2013). SIRT1 participates not only in the regulation of the cell cycle by deacetylating histones, but also in positively or negatively regulating downstream targets that play key roles in various physiological activities, such as oxidative/antioxidant homeostasis, inflammation, and energy metabolism (Figure 1). For example, adenosine 5′-monophosphate (AMP)-activated protein kinase (AMPK) is an AMP-dependent, essential kinase that regulates energy homeostasis. The AMPK and SIRT1 signaling pathways are evolutionarily conserved energy sensors; AMPK senses changes in the cellular AMP/ATP ratio, and SIRT1 senses changes in the NAD+/NADH ratio (Salminen and Kaarniranta, 2012). AMPK enhances SIRT1 activity by increasing the NAD + concentration; in turn, SIRT1 activation can promote AMPK activity (Cantó et al., 2009). The important function of the AMPK/SIRT1 signaling pathway in regulating autophagy, anti-inflammatory, and antioxidant activities has been studied extensively. The activation of the AMPK/SIRT1 signaling pathway by drugs to treat metabolic and aging-related diseases is also a current research topic (Maharajan and Cho, 2021; Wang et al., 2021). Peroxisome proliferator-activated receptor gamma coactivator 1-alpha (PGC-1α), the downstream factor of SIRT1, is involved in the regulation of many cellular physiological functions, including the control of mitochondrial homeostasis and reactive oxygen species (ROS) levels (Johri et al., 2013), and activation of PGC-1α requires deacetylation of SIRT1 and dephosphorylation of AMPK (Kaarniranta et al., 2018; Xu et al., 2021). Currently, aging is accompanied by a decrease in autophagic efficiency (Salminen and Kaarniranta, 2009; Barnes et al., 2019). SIRT1 can regulate the level of autophagy by deacetylating p53, which has been shown to inhibit autophagy when located in the cytoplasm but promote autophagy when located in the nucleus (Kroemer et al., 2010). This observation is consistent with the idea that SIRT1 has different cellular localizations at different stages of the cell cycle. SIRT1 is transferred from the nucleus to the cytoplasm in senescent cells, thus decreasing autophagy. SIRT1 also deacetylates forkhead box Os (FoXOs), enhancing cellular resistance to oxidative stress (Singh and Ubaid, 2020). Moreover, nuclear factor kappa B (NF-κB), a key regulator of inflammation, can be inhibited by SIRT1, reducing the expression of inflammatory factors and thus acting as an anti-inflammatory agent (de Gregorio et al., 2020). There is growing evidence that microRNAs, small non-coding single-stranded RNAs, are involved in SIRT1 regulation, and regulating the expression levels of specific miRNAs may be a new therapeutic strategy (Munk et al., 2017; Barnes et al., 2019).

## Current studies on silent information regulator 1 and age-related hearing loss

A search of PUBMED for the keywords “SIRT1,” “hearing loss,” “cochlea,” “presbycusis” and “age-related hearing loss,” showed that there were less than 50 studies related to SIRT1 and hearing until now, of which 14 studies (Table 1) were related to ARHL.

**TABLE 1:** SIRT1 and ARHL related articles.

Subject	Treatment	Result	Author
SAMP1mice and HepG2 cells	Ubiquinol-10	Ubiquinol-10 may enhance mitochondrial activity by increasing levels of SIRT1, PGC-1α, and SIRT3 that slow the rate of ARHL and protect against the progression of aging and symptoms of age-related diseases.	Tian et al., 2014
C57BL/6 mice	None	It was confirmed that SIRT1 exists in cochlea and auditory cortex and decreases with age.	Xiong et al., 2014
HEI-OC1 auditory cells and C57BL/6 mice	miR-34a overexpression and knockdown, resveratrol	miR-34a overexpression was inhibited SIRT1, while resveratrol can activate SIRT1 and improve hair cell death and hearing loss.	Xiong et al., 2015
CBA/J mice	None	In the cochlea, the expression of SIRT1, 3, and 5 (both mRNA and protein) was decreased in the old mice, whereas no noticeable difference was observed regarding SIRT2, 4, 6, or 7.	Takumida et al., 2016
C57BL/6 mice and human blood	None	Circulating miR-34a levels in mice and humans correlated with age-related hearing loss, but SIRT1 did not correlate with human ARHL.	Pang et al., 2016
C57BL/6 mice and HEI-OC1 auditory cells	SIRT1 knockdown, hydrogen peroxide	SIRT1 deficiency activated Foxo3a, increased cochlear hair cell peroxidase activity, and SIRT1 knockdown mice delayed the onset of ARHL.	Han et al., 2016
C57BL/6 mice and HEI-OC1 auditory cells	H2O2, overexpression of miR-29b, transfection with the miR-29b inhibitor	miR-29b/SIRT1/PGC-1α signaling was involved in the development and progression of ARHL, miR-29b modulated mitochondrial dysfunction and apoptosis through SIRT1/PGC-1α signaling in HEI-OC1 cells.	Xue et al., 2016
C57BL/6 mice, human blood, SK-N-MC and SH-SY5Y cells	H2O2, MIAT (myocardial infarction associated transcript),transfected with anti-miR-29b	Relative expression of MIAT, SIRT1 and PGC-1α was downregulated in aged mice, with microRNA-29b (miR-29b) being highly expressed. MIAT binds to miR-29b, an inhibitor of SIRT1 expression.	Hao et al., 2019
C57BL/6 mice and HEI-OC1 auditory cells	Resveratrol, SRT1720	Long-term resveratrol feeding increased SIRT1 expression in hair cells of aged mouse cochlea, improved autophagy in outer hair cells, attenuated ARHL.	Pang et al., 2019
C57BL/6 mice and HEI-OC1 auditory cells	Resveratrol, SIRT1 overexpression, miR-34a knockdown	Long-term resveratrol feeding improved the balance of mitochondriogenesis and mitochondrial autophagy in the mouse cochlea and the miR-34a/SIRT1 signaling pathway was involved in delaying ARHL.	Xiong et al., 2019
C57BL/6 mice	P43 knockdown	P43-/-mice decreased SIRT1 expression, altered mitochondrial morphology and function, and increased oxidative stress and apoptosis, which aggravated ARHL.	Affortit et al., 2021
Sprague-Dawley rats	Environmental enrichment	Exposure to EE for 12 weeks resulted in activation of the central auditory pathway and limbic system SIRT1 in rats, reduced chronic inflammation, and improved ARHL.	Song et al., 2021
C57BL/6 mice	High-fat diet (HFD), N1-methylnicotinamide (MNAM)	High-fat diet reduced SIRT1 levels in the cochlea and aggravates ARHL, while MVAM increased SIRT1 levels and attenuated ARHL.	Miwa, 2021
C57BL/6 mice	Thymoquinone (TQ)	TQ activated SIRT1, reduced cilia damage of hair cells, and improved ARHL	Salam et al., 2021

In 2013, this is the first study to demonstrate the presence of SIRT1 in the cochlea and auditory cortex, which was significantly expressed in the nuclei of inner and outer hair cells, vascular striated marginal and basal cells, spiral ligament fibroblasts, and spiral ganglion cells and decreased with age (Xiong et al., 2014). Overexpression of miR-34a inhibited SIRT1 with increased apoptosis. After resveratrol administration, miR-34a could be inhibited and the apoptosis of hair cells was reduced, as well as the threshold drift in mice (Xiong et al., 2015). Later, SIRT1 overexpression in mice by the transgenic technique confirmed that overexpression of SIRT1 could protect cochlear hair cells and improve ARHL (Xiong et al., 2019). In contrast, one study in 2016 suggested that SIRT1 deficiency activated Foxo3a, enhanced the resistance of cochlear hair cells to oxidative stress damage, and delayed the occurrence of ARHL (Han et al., 2016). However, the results of several relevant studies in recent years suggest the opposite of this conclusion.

In 2019, researchers compared 2- and 12-month-old C57BL/6 mice, showing that SIRT1 expression was significantly decreased in 12-month-old mice. However, resveratrol, a natural SIRT1 activator, was added to the diet, and the mice were fed for a total of 10 months, beginning at ∼2 months of age. Compared with the conventionally fed mice of the same age, the supplementation of resveratrol increased the SIRT1 level in the outer hair cells of the cochlea of mice and induced autophagy and significantly improved the ARHL (Pang et al., 2019).

The normal and orderly physiological activities cannot be separated from the normal functioning of mitochondria (Rossmann et al., 2021). P43, as the mitochondrial receptor of T3 (Wrutniak et al., 1995), is closely related to mitochondrial function (Grandemange et al., 2005). It was found that mice with selective knockout of the P43 gene did not exhibit hearing loss at a young age, but compared with wild-type C57BL/6J mice, p43 knockout mice (p43-/-) showed an earlier increase in hearing threshold and more severe hearing loss with age and exhibited greater sensitivity to noise damage. The authors suggested that this may be related to the altered mitochondrial morphology and function in p43-/- mice, the dramatic decrease in SIRT1 activity and Bcl-2 expression, and the subsequent increase in oxidative stress, inflammation and apoptosis (Affortit et al., 2021). There may be a direct causal relationship between decreased SIRT1 activity and earlier onset and more severe ARHL, which needs to be further verified using subsequent treatments such as SIRT1 activators, inhibitors or transgenics.

In 2021, another study exposed SD rats to environmental enrichment (EE) for 12 weeks, where the animals were placed in cages full of toys and encouraged to forage and explore by moving the toys, feeding boxes and water tanks at least once a week. After 12 weeks, they found that EE increased SIRT1 activity in the auditory cortex and improved ARHL (Song et al., 2021). A high-fat diet reduced cochlear SIRT1 and aggravated ARHL, while the addition of MNAM (N1-methylnicotinamide) elevated SIRT1 levels and inhibited ARHL (Miwa, 2021). These results show that adequate SIRT1 expression plays a key role in ARHL. The study of SIRT1 as a target for ARHL and the use of drug activation of SIRT1 to prevent or medicate ARHL are hot spots for future research.

Since the acquisition of ARHL in animal models is time-consuming, it increases the difficulty of the experiment, while the modeling time of noise-induced and drug-induced deafness is fast, and the modeling effect is stable. At present, there are many studies on cisplatin-induced hearing loss and noise-induced deafness. The protective effect of SIRT1 in cisplatin-induced hearing loss and noise-induced deafness confirms the potential therapeutic role of SIRT1 in the cochlea (Pang et al., 2018; Chen X. M. et al., 2020; Zhan et al., 2021; Liu et al., 2022). To obtain the cytotoxic or cytoprotective effect of a certain treatment on hearing in a short time, experiments *in vitro* are preferred. The HEI-OC1 cell line, one of the auditory cell lines, is now commonly used in hearing research (Kang et al., 2020; Zheng et al., 2020; Zhang X. S. et al., 2021). Cochlear explants or basilar membranes cultured *in vitro* have also been used to study the effect of drugs on hearing (He et al., 2020). However, experiments *in vitro* do not reflect the systemic effects of a treatment as *in vivo* do, and administration *in vivo* may not work well in the inner ear because of the blood-vagus barrier of the inner ear (Nyberg et al., 2019), leading to different results after *in vivo* and *in vitro* administration, which is a challenge that needs to be addressed. Hearing loss is not only determined by hair cells but is also closely related to spiral ganglia, spiral ligaments, vascular striae, and endolymph (Cunningham and Tucci, 2017; Korver et al., 2017). The *in vitro* cell assays provide information about the cellular response to the treatment and can be used to understand drug effects on hair cells, but they are not representative of drug effects on hearing.

## Prospects

Age-related hearing loss is a disease associated with aging, the exact cause of which is unknown and for which there is no clear and effective treatment. Caloric restriction, currently recognized as an effective measure to delay aging, can alter NAD+/NADH and then activate SIRT1 (Madeo et al., 2019). Perhaps the prevention and treatment of ARHL needs to be carried out on many fronts, including life, diet and medication. Studies have confirmed that SIRT1 plays a highly important role in ARHL as well as aging, tumors, and neurodegenerative diseases by acting on downstream signaling molecules such as p53 and NF-κB to mitigate DNA damage, maintain the oxidative/antioxidant balance, and reduce apoptosis. Moreover, a number of drugs have been shown to activate SIRT1, which has positive anti-aging, anti-inflammatory and antioxidant effects. However, these studies are currently limited to animal or cellular experiments. A large number of clinical studies are needed to clarify whether SIRT1 plays the same positive role in the human cochlea, whether these drugs can be safely used in humans and the timing of interventions to prevent ARHL.

## Author contributions

TZ wrote the manuscript and prepared all the figure and table. GT supervised the manuscript writing and editing. Both authors contributed to the article and approved the submitted version.

## Abbreviations

SIRT1: silent information regulator 1
ARHL: age-related hearing loss
NAD+: nicotinamide adenine dinucleotide
Sir2: silent information regulator 2
AMPK: AMP-activated protein kinase
PGC-1α: peroxisome proliferator-activated receptor gamma coactivator 1-alpha
FOXO: forkhead box O
NF-κB: nuclear factor kappa B
ROS: reactive oxygen species
G6PD: glucose-6-phosphate dehydrogenase.
