# Corrigendum: Potential therapeutic role of SIRT1 in age- related hearing loss

**DOI:** 10.3389/fnmol.2022.1099324

**Published:** 2022-12-05

**Authors:** Tingting Zhao, Guangyong Tian

**Affiliations:** ^1^Department of Otorhinolaryngology-Head and Neck Surgery, The Third Affiliated Hospital of Southern Medical University, Guangzhou, China; ^2^Guangdong Provincial Key Laboratory of Bone and Joint Degeneration Diseases, Guangzhou, China

**Keywords:** SIRT1, age-related hearing loss, ARHL, cochlea, hearing loss, age

In the published article, there was an error in the article title. Instead of “STRT1,” it should be “SIRT1.”

The authors apologize for this error and state that this does not change the scientific conclusions of the article in any way. The original article has been updated.

